# Individual Plasticity of the Shade Response of the Invasive *Solidago canadensis* in China

**DOI:** 10.1371/journal.pone.0170049

**Published:** 2017-01-12

**Authors:** Leshan Du, Haiyan Liu, Ming Yan, Junmin Li, Junsheng Li

**Affiliations:** 1 Research Center for Biodiversity, Chinese Research Academy of Environmental Sciences, Beijing, Beijing, China; 2 Zhejiang Provincial Key Laboratory of Evolutionary Ecology and Conservation, Taizhou University, Taizhou, Zhejiang, China; 3 School of Life Science, Shanxi Normal University, Linfen, Shanxi, China; Eidgenossische Forschungsanstalt fur Wald Schnee und Landschaft Institut fur Schnee- und Lawinenforschung, SWITZERLAND

## Abstract

To evaluate the population variation, individual plasticity, and local adaptability of *Solidago canadensis* in response to shade treatment, we conducted a common pots experiment with a total of 150 ramets (5 genets, 15 populations, and 2 treatments) subjected to both control (natural light) and shady treatment (10% of natural light). Shade treatment significantly reduced growth and content of defense metabolites in *S*. *canadensis*. Compared to control, shading led to increased height, decreased basal diameter, increased leaf width, increased leaf length, increased chlorophyll content, stronger photosynthetic rate (*P*_n_), stronger stomatal conductance (*g*_s_), and lower root to shoot ratio. Three-way analysis of variance revealed geographical origin to significantly affect the basal diameter of *S*. *canadensis*, while genotype significantly affected plant height, intercelluar CO_2_ concentration (*C*_i_), transpiration rate (*T*_r_), and proline content. Significant interactive effects between shade and geographic origin were prevalent for most traits. The phenotypic differentiation coefficient of the plasticity of all traits was below 0.4, indicating that most of all variations can be found among individuals within populations. Phenotypic selection analysis revealed that fitness was significantly positively related to plant height, basal diameter, *C*_i_, total flavonoid content, as well as the plasticity of plant height, leaf length, leaf width, *g*_s_, *C*_i_, total flavonoid content, and malondialdehyde content under the control condition. However, subjected to shade, fitness was only significantly positively related to plant height, basal diameter, and the plasticity of basal diameter. Rather than local adaption, these results suggest that individual plasticity played a more prominent role in the shade response of the invasive *S*. *canadensis*.

## Introduction

A growing consensus agrees that the environment has the ability to induce changes in the behavior of individual plants at a morphological and, or physiological level and that such changes might be crucial for survival in heterogeneous and variable conditions [1]. Functionally adaptive plasticity in allocational, morphological, and physiological traits that are involved in resource acquisition can allow individual plants to maximize their reproductive fitness in diverse environments. Plasticity plays a central role in biological invasions via allowing individuals to colonize environmentally diverse habitats and establish healthy populations [2, 3]. Furthermore, an invasive species may also spread across diverse habitats via local adaptation, evolving ecotypes with distinctive traits, and patterns of individual plasticity [4–7]. Although the majority of all published studies showed that both phenotypic plasticity and local adaptation contributed to invasion success [5, 8], the relative importance of both strategies has been subject to debate [9] and depends on species. Matesanz et al. verified that phenotypic plasticity (rather than locally adapted ecotypes) allows the Asian annual plant *Polygonum cespitosum* to colonize a wide range of habitats in northeastern North America [10].

Heterogeneous light environment is a dominant stress factor for plants and light is an extremely heterogeneous environmental factor, particularly in disturbed sites [11, 12]. Plants respond to changing light conditions by adjusting a series of morphological and physiological traits, such as leaf size, specific leaf area, net photosynthetic, and patterns of biomass allocation [13–15]. When confronted with low light intensities, invasive plants partitioned more area and biomass to leaves to increase light capture efficiency and improve light utilization efficiency to maximize carbon gain. Different responses to light can affect the process of invading and spreading, and further alter the competitive relationship between invasive and native plants. Consequently, the dynamics of population and community of invasive and native plants can be drastically altered.

*Solidago canadensis* L. (Asteraceae) is a perennial herb native to North America and was first introduced to Shanghai, China as an ornamental in 1935 [16]. Currently, it is widely distributed along the southeast coastal and the Yangtze River basin, and is also a major invasive plant species in many other countries [17, 18]. *S*. *canadensis* can severely damage local biodiversity, and the natural ecosystem. Furthermore, the very effective sexual and asexual reproduction can cause enormous economic losses. Several studies found that shade decreased growth and photosynthetic ability [19]. However, to the best of our knowledge, population variation of the phenotypic plasticity in response to shade has not been explored in *S*. *canadensis*. We hypothesized that different geographical populations would show different adaptive responses to shade. Our goal was to reveal the traits preferred by plants that thrive under shade and whether individual plasticity or local adaption plays the more important role in invasive species. Understanding the shade response of *S*. *canadensis* will help to predict invasive trends, and moreover provide basic references for the management and prevention of the invasive *S*. *canadensis*.

## Materials and Methods

### Plant sampling and propagation

In October 2012, rhizomes of *S*. *canadensis* were collected from 15 populations with a range of latitudes from 26.0968°N to 34.654°N and longitudes from 111.532°E to 121.804°E (Table 1). Within every population, 12 randomly selected ramets were dug out. The distances between collected rhizomes were at least 10 m to reduce the probability of sampling the same genet more than once. Our field studies did not involve any endangered or protected species and none of the population were privately owned or under nature protection. No specific permissions were required for these locations. Shoot bases with attached rhizomes were transferred to the Taizhou University in Linhai City, Zhejiang Province, China and kept moist until replanting. Rhizomes were individually planted in pots with diameters of 30 cm and depths of 30 cm. The soil mixture (yellow clay soil: sand: peat soil = 6:3:1) had a final pH of 6.80 ± 0.10, an organic matter content of 27.66 ± 0.69 g kg^−1^, a total nitrogen content of 361.00 ± 19.05 mg kg^−1^, an available phosphorus content of 8.00 ± 0.66 mg kg^−1^, and an available potassium content of 12.00 ± 0.58 mg kg^−1^. All plant material used in this study were propagated twice in a greenhouse for two years and three months and the environmental carryover effects were minimized.

**Table 1:** Locations and habitats of 15 *S*. *canadensis* populations.

No.	Population abbreviation	Location	Longitude	Latitude	Altitude (m)	Habitats
1	FZ	Fuzhou City, Fujian Province	N119.359°	E26.098°	19	Abandoned farmland
2	WZ	Wenzhou City, Zhejiang Province	N120.607°	E28.126°	4	Abandoned farmland
3	TZ	Taizhou City, Zhejiang Province	N121.397°	E28.656°	6	Abandoned farmland
4	JDZ	Jingdezheng City, Jiangxi Province	N117.166°	E29.318°	40	Green belts
5	JJ	Jiujiang City, Jiangxi Province	N116.283°	E29.985°	18	Abandoned vegetable garden
6	HZ	Xiaoshan District, Hangzhou City, Zhejiang Province	N120.297°	E30.161°	9	Abandoned farmland
7	WC	Wuchang District, Wuhan City, Hubei Province	N114.421°	E30.541°	26	Abandoned vegetable garden
8	YC	Yichang City, Hubei Province	N111.532°	E30.843°	333	Abandoned building land
9	WH	Hankou District, Wuhan City, Hubei Province	N114.350°	E30.878°	25	Abandoned farmland
10	HQ	Minhang District, Shanghai City	N121.433°	E31.307°	5	Green belts
11	WHu	Wuhu City, Anhui Province	N118.387°	E31.342°	16	Garbage dump
12	PD	Pudong District, Shanghai City	N121.804°	E31.354°	3	Abandoned farmland
13	NJ	Nanjing City, Jiangsu Province	N119.094°	E31.794°	22	Abandoned farmland
14	LYG	Lianyungang City, Jiangsu Province	N19.235°	E34.654°	3	Abandoned farmland
15	NT	Nantong City, Jiangsu Province	N120.843°	E32.070°	5	Abandoned farmland

### Experiment design

In June 2014, newly emerged *S*. *canadensis* ramets with similar height (approximately 15 cm) were cut off from their respective stock and individually planted in pots with diameters of 16 cm and depths of 14 cm filled with soil mixture mentioned above. A randomly chosen subset of pots was moved inside a shed covered with white nylon anti-fly net.

After 14 days, we recorded plant height (H_t1_) and the number of leaves (N_t1_) of *S*. *canadensis* and initiated the shade experiment. Two ramets from each of five genets of the 15 *S*. *canadensis* populations (in total 150 ramets, one replicate per genotype and per treatment) were allocated to one of two treatments: a control and a shade treatment. In the control treatment, plants were cultured under natural light. In the shade treatment, light availability was reduced by 90% via shading the plants with double-black and semi-transparent nylon nets clamped to a mental frame at 150 cm height [20]. Light intensities were measured via a handheld luxmeter. The positions of the pots were randomly changed every week to reduce position bias effects. No fertilizer was used and an adequate amount of water was provided.

### Measurements

Sixty days after transplanting, plant height (H_t2_), leaf length, and leaf width were measured via ruler with an accuracy of 0.1 cm and the leaf length/width ratio was calculated. The number of leaves (N_t2_) was also recorded. The basal diameter was measured via vernier caliper with an accuracy of 0.02 cm. The rate of increase of the number of leaves was calculated as (N_t2_-N_t1_)/(t_2_-t_1_), and the increase rate in plant height was calculated as (H_t2_-H_t1_)/(t_2_-t_1_).

*In situ* photosynthesis measurements were made on the third fully expanded leaf, counted from the tip of the shoot and using a portable photosynthesis-measurement system (LI-6400 XT, Li-COR Inc., Lincoln, NE, USA). Measurements were obtained between 9:00 AM and 11:00 AM under a photosynthetically active radiation of 1,400 μmol m^-2^ s^-1^ (i.e. at light saturation) at a leaf temperature of 25°C, a CO_2_ concentration of 400 ppm, and relative humidity of 70%. Leaf area of effective photosynthesis was traced via leaf area analyzer (Win FOLIA, Regent Instruments Inc). Net photosynthetic rate (*P*_n_), stomatal conductance (*g*_s_), transpiration rate (*T*_r_), and intercellular CO_2_ concentration (*C*_i_) were measured.

Three tender leaves per plant were collected and transferred to the lab immediately upon collection. Proline content was measured via acidic ninhydrin colorimetry [21]. The malondialdehyde and soluble sugar contents were measured via the thiobarbituric acid (TBA) method [22]. The chlorophyll content was measured spectrophotometrically following procedure published by Wintermans and De Mots [23]. All required weight was measured via balance with an accuracy of 0.1 mg.

Three matured leaves per plant at a similar position were collected and transferred to the lab immediately upon collection. The leaves were dried at 70°C and ground into powder. Experimental weight of each leaf was weighted via balance with an accuracy of 0.1 mg. Flavonoid content was measured via the rutin method [24]. The total phenolic content was determined via the Folin-phenol method [25]. Lignin content was measured via concentrated sulfuric acid titration [26].

Following measurements, plants were harvested and divided into leaves, stems, and roots. Plant material was over-dried in an oven (at 105°C for 1 h and then at 80°C until a constant weight was reached). The leaf, stem, and root biomasses were weighed via balance with an accuracy of 0.1 mg. Total biomass and root/shoot ratios were calculated.

### Statistical analysis

Paired t-test was used to analyze the difference in the rate of increase in the number of leaves, in plant height, and biomass traits between the shade and control treatment. Three-way ANOVA was conducted using treatment as fixed factor, population as random factor, and genotype as a random factor nested within population. Plant height at the onset of the experiment was used as a covariation to exclude the initial difference effect among different plants. All data is normally distributed, and satisfies homogeneity of variance.

The phenotypic plasticity index (PPI) was calculated as: (max(X_0_, X_i_)—min(X_0_, X_i_)) / max(X_0_, X_i_), where X_0_ and X_i_ stand for the mean values of control and shade treatments, respectively. Max(X_0_, X_i_) is the higher value of X_0_ and X_i_, and min(X_0_, X_i_) the lower value of X_0_ and X_i_ [26, 27]. The mean PPI per trait was calculated via the PPI value of 15 populations. The phenotypic differentiation coefficient (*Vst*) was calculated as: *Vst* = (δ^2^_t/s_) / (δ^2^_t/s_ + δ^2^_s_), where δ^2^_t/s_ and δ^2^_s_ stand for the variance components within and among population, respectively [28].

To test whether phenotypic traits and plasticity of traits responded to the shade treatment increase the fitness of a genotype (j), we regressed the fitness value (biomass, *W*_j_) of a genotype across both environments against the trait value (X_j_) across both environments as well as the plasticity (*P*_j_), using the following equation Wj = Constant + α*X*_j_ + β*P*_j_ [29,30].

All statistical analyses were conducted using SPSS 18.0 (IBM Co., USA) and all figures were plotted using Origin 9.0 (OriginLab Co., USA).

## Results

### Shade response of *S. canadensis*

**Fig 1:**
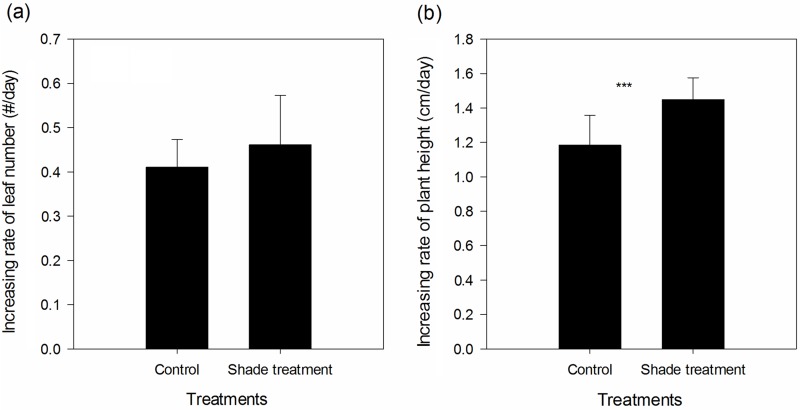
Effect of shade treatment on the increase rate of the number of leaves (a) and plant height (b) of *S*. *canadensis*. *** indicates a significant difference between control and the shade treatment at *p* < 0.001.

**Fig 2:**
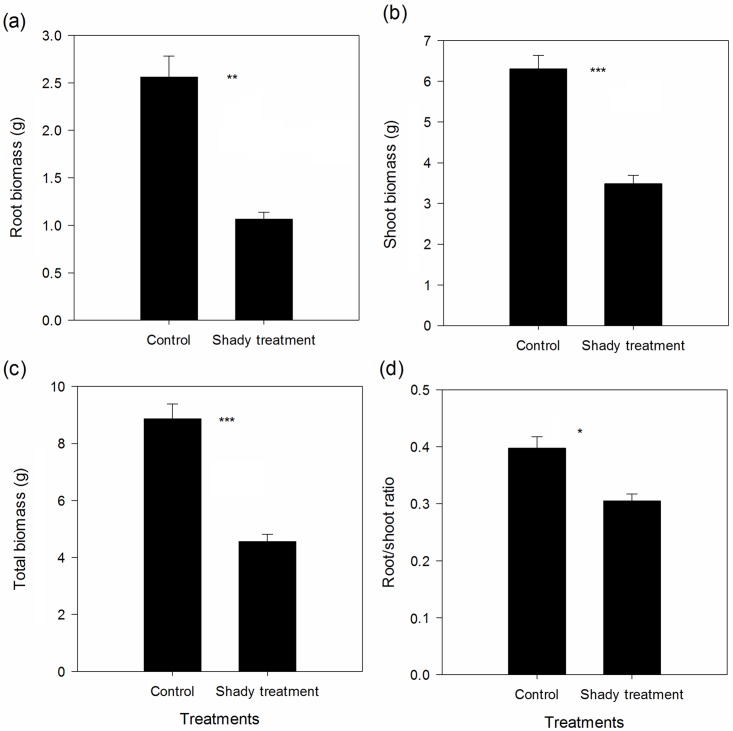
Effect of shade treatment on root biomass (a), shoot biomass (b), total biomass (c), and root/shoot ratio (d) of *S*. *canadensis*. *, ***, indicate significant differences between control and shade treatment at *p* < 0.05 and *p* < 0.001, respectively.

Shade treatment significantly elevated the rate of plant height increase (Fig 1B, paired *t* value = -5.059, *p* < 0.001); however, shade treatment had no significant effect on the rate of increase of the number of leaves (Fig 1A, paired *t* value = -1.867, *p* = 0.083). Shade treatment significantly reduced root biomass (Fig 2A, paired *t* value = 6.807, *p* < 0.001), shoot biomass (Fig 2B, paired *t* value = 10.785, *p* < 0.001), total biomass (Fig 2C, paired *t* value = 10.832, *p* < 0.001), and root/shoot ratio (Fig 2D, paired *t* value = 3.274, *p* = 0.006).

### Phenotypic plasticity of *S. canadensis*

The two-way ANOVA revealed that shade had a significant effect on the morphological, physiological, and defense traits of *S*. *canadensis* with the exception of *C*i and *T*r (Table 2). Shade significantly increased plant height, leaf length, leaf width, chlorophyll content, *P*n, and *G*s, whereas basal diameter, proline content, malondialdehyde, soluble sugar, total phenolic, total flavonoids, and lignin contents were significantly decreased. Geographical origin only significantly affected the basal diameter of *S*. *canadensis* (*p* > 0.05). Genotype significantly affected plant height, *C*i, *T*r, and proline content (*p* < 0.05 for each). Significant interactive effects between shade and geographic origin were revealed for plant height, leaf length, leaf width, basal diameter, chlorophyll content, *P*n, total flavonoid content, total lignin content, and proline content (*p* < 0.05) (see Table 2).

**Table 2:** Phenotypic traits in control treatment and shade treatment as well as phenotypic plasticity indexes (PPI) of each measured trait of *S*. *canadensis*. The data were shown as means ± standard error (SE). Two-way ANOVA results are listed.

Trait	Mean±SE		Two-way ACNOVA				Plasticity	
	Control	Shady	*F*_shady_	*F*_origin_	*F*_genotype_	*F*_shady×origin_	Mean±SE	*V*st
Plant height (cm)	66.81±1.87	79.74±1.77	25.86**	0.7	3.25**	2.16*	0.20±0.02	0.04
Leaf length(cm)	9.26±0.22	11.05±0.31	14.47**	0.76	1.65	2.66**	0.19±0.02	0.02
Leaf width(cm)	1.61±0.05	2.17±0.07	26.42**	0.64	1.29	2.17*	0.27±0.02	0.02
Basal diameter(cm)	0.75±0.01	0.58±0.01	79.05**	2.79*	1.46	2.56**	0.23±0.01	0.02
Chlorophyll content (mg/g)	8.51±0.26	27.61±0.51	670.45**	2.01	1.04	2.59**	0.68±0.01	0.09
Photosythetic rate (μmol·m^-2^·s^-1^)	9.56±0.39	12.61±0.25	37.09**	1.04	1.76	2.09*	0.27±0.03	0.21
Stomatal conductance (mol·m^-2^·s^-1^)	0.5±0.02	1.26±0.13	29.48**	1.22	1.29	1.51	0.51±0.03	0.25
Intercelluar CO_2_ concentration (μmol·mol^-1^)	337.29±2.56	342.2±3.85	1.51	1.29	1.86*	1.18	0.05±0.01	0.29
Transpiration rate (mmol·m^-2^·s^-1^)	9.74±0.33	9.42±0.32	0.76	1.96	2.67**	1.73	0.17±0.02	0.31
Total phenolic content (mg/g)	36.31±0.84	8.83±0.43	734.56**	1.54	1.36	1.97	0.75±0.01	0.36
Total flavonoids content (mg/g)	0.51±0.02	0.16±0.01	140.5**	1.36	1.25	2.41*	0.68±0.02	0.38
Total lignin content(mg/g)	3.12±0.14	1.51±0.11	34.96**	0.81	0.97	3.47**	0.50±0.03	0.18
Proline content (ug/g)	79.79±3.57	62.41±1.93	13.26**	1.29	1.7*	2.33**	0.26±0.02	0.13
Malondialdehyde content (mmol/g)	0.06±0	0.05±0	13.12**	0.9	0.93	1.59	0.20±0.02	0.02
Soluble sugar content (mg/g)	61.05±3.26	44.59±2.52	12.65**	1.59	0.95	1.72	0.34±0.03	0.13

Note:

*, ** indicate significant differences at *p* < 0.05 and *p* < 0.01 level.

The phenotypic plasticity indices of chlorophyll content, *C*i, total phenolic content, total flavonoid content, and total lignin content were all above 0.4, while those of leaf length, *C*i, and *T*r were below 0.2 (Table 2). The phenotypic differentiation coefficiencies of the plasticity of all the traits were below 0.4, indicating that most of the variation of phenotypic plasticity existed within populations of different geographical origins, not among populations (Table 2).

### Phenotypic selection of shade

Phenotypic selection analysis showed that fitness significantly and positively correlated with plant height, basal diameter, intercellular CO_2_ concentration, total flavonoid content, proline content, as well as the plasticities of plant height, leaf length, leaf width, stomatal conductance, intercellular CO_2_ concentration, total flavonoid content, and malondialdehyde content under normal condition. However, under shade treatment, significantly and positively correlations were only detected for plant height, basal diameter, and the plasticity of basal diameter (Table 3).

**Table 3:** Standardized regression coefficients of fitness metrics of traits (α) and plasticity of traits (β) under control and shade treatment. All values are based on genotypic values and fitness was measured as biomass. Benefit of traits and plasticity of traits are indicated via positive regression coefficients.

Traits	Control		Shade treatment	
	α value	β value	α value	β value
Plant height	0.967***	0.330***	0.789***	-0.024
Leaf length	0.025	0.306**	0.069	0.139
Leaf width	0.094	0.251*	0.090	0.069
Basal diameter	0.381**	0.008	0.496***	0.229*
Chlorophyll content	-0.069	0.050	-0.170	0.109
Photosythetic rate	0.105	0.252	0.013	-0.007
Stomatal conductance	0.258	0.371**	-0.035	0.055
Intercelluar CO_2_ concentration	0.271*	0.529***	0.642	0.641
Transpiration rate	0.043	0.153	0.015	-0.029
Total phenolic content	0.245	0.265	0.014	-0.057
Total flavonoids content	0.368*	0.398*	-0.008	-0.004
Total lignin content	0.170	-0.025	0.286	0.216
Proline content	0.777**	-0.044	-0.010	0.187
Malondialdehyde content	0.036	0.321**	-0.040	0.093
Soluble sugar content	-0.093	0.200	0.189	0.114

Note:

*, **, and *** indicate significant differences at *p* < 0.05, *p* < 0.01 and *p* < 0.001 level.

## Discussion

Previous studies have reported a detrimental effect of shade on plants, especially affecting anatomical structure, phenotypic, and physiological traits, hence reducing fitness traits related to biomass [1, 27]. We found similar results in our study. Shade treatment evidently decreased root biomass, shoot biomass, and total biomass of *S*. *canadensis*. These results suggest that shade treatment significantly reduced plant fitness, suggesting that low light intensity might not be a suitable habitat for *S*. *canadensis*. This may explain why the main distribution habitats of *S*. *canadensis* in China are open fields, rather than habitats with shady conditions [19].

Different light intensities significantly changed the morphological and physiological traits of plants [28]. In this study, we found *S*. *canadensis* to express the typical shade-avoidance syndrome [29], i.e. increased height and decreased basal diameter, reducing the mutual shade effect. Furthermore, increased width and length of leaves enable the interception of more light; increased chlorophyll content improves utilization efficiency, and stronger photosynthetic rate and stomatal conductance aid in the absorbance of light resources when exposed to shade. Similar results were reported enabling plants to functionally accommodate low light conditions and consequently, plants express developmental modifications that maximize light interception, e.g. via increasing specific leaf area, leading to large, thin leaves and elongating seedling internodes and stems, all of which facilitates the escape from a low-light environment for *S*. *canadensis* [27, 30–33]. Interestingly, an enhanced ability of light absorption and utilization efficiency, coupled with lower biomass was found in the shady treatment. This suggests that a complementary effect of the shady responses could not complement the inhibitory effect of shade on *S*. *canadensis* and 10% light intensity was a limiting factor for *S*. *canadensis*. We also found that shade significantly reduced the root/shoot ratio, indicating an adaptive strategy to allocate more energy to aboveground biomass to improve fitness and competitive ability [34]. In this study, we also found that shade significantly decreased the contents of soluble sugar, total phenolic compounds, total flavonoids, and lignin. In addition, the content of proline and malondialdehyde were also decreased, indicating that the anti-oxidant ability of *S*. *canadensis* reduced when exposed to shade. *S*. *canadensis* reduced resource allocation to defense mechanisms and anti-oxidant metabolites to allocate more energy to essential organs, thus increasing the growth input and retaining fitness in harsh condition. Shade tolerance is one of the most important ecological factors with respect to interspecific competition [35]. Thus, the shade tolerance ability of *S*. *canadensis* might play a key role for the successful invasion of this species.

The mean PPI of *S*. *canadensis* in response to shade was 0.36, with minimal intercelluar CO_2_ concentration (0.05) and maximal chlorophyll and total flavonoid contents (0.68). Matos et al. reported that plasticity of physiological and biochemical traits in *Coffea arabica* is more important for acclimation to intra-canopy light variations than morphological or anatomical trait plasticity [36]. In this study, we found that the mean PPI of defense traits was 0.455, which was above that of morphological traits (mean 0.223) and photosynthetic physiological traits (mean 0.336). These results suggest that photosynthetic physiological traits and defense traits were easier to change in response to shade and more important for the successful propagation of *S*. *canadensis*.

In this study, geographical origin only significantly affected the basal diameter of *S*. *canadensis*, not any other traits. However, we found significant interactive effect between shade and geographic origin for most of the traits of *S*. *canadensis*, indicating that the effect of shade varied among different populations. Si et al. reported that the effect of geographical origin and interactions between geographical origins and light treatments were all significant in traits of the invasive *Wedelia trilobat* and the authors thus concluded that both phenotypic plasticity and local adaptation occurred in *W*. *trilobata* in response to the light condition [9]. Adaptation to full sunlight intensity was also detected in *W*. *trilobata* populations from sunny sites, while this was not detected for those grown in shady sites [9]. All populations in our study were collected from typical *S*. *canadensis* habitats near the roadside, abandoned fields, and similar habitats (open field). All the populations grew in a full sunlight habitat without any shade and most of the accompanied species were grasses and small shrubs. Phenotypic selection analysis revealed only significantly positively relationships for plant height, basal diameter, and the plasticity of basal diameter under shade treatment. However, the phenotypic differentiation coefficient of plant height and basal diameter were only 0.04 and 0.02, respectively, indicating most of the variation existed among individuals within populations, rendering the existence of local adaption impossible. In addition, genotypes from all populations significantly affected the traits of height, *C*_i_, *T*_r_, and proline content, indicating individual plasticity rather than local adaption played central roles in the shade response of *S*. *canadensis*. The lack of local adaptation might be a result of a similarity of habitats of *S*. *canadensis* in China, without light heterogeneity. Further studies, investigating more populations from more variable habitats, should be conducted to reveal a general rule of the shade response of *S*. *canadensi*s.

## Conclusion

Shade evidently altered phenotypic and physiological traits, and furthermore reduced plant fitness, i.e. biomass of *S*. *canadensis*. However, typical shade-avoidance syndromes were also found in our study, such as increased height, increased width and length of leaves, increased chlorophyll content, and decreased root/shoot ratio, which all might play central roles in a successful invasion. Furthermore, photosynthetic physiological traits and defense traits changed in response to shade. Our study suggests that individual plasticity rather than local adaption played the key role in the shade response of *S*. *canadensis*. These adaptive strategies of S. canadensis to endure light deficiency provide a basic reference for the management and control of this invasive species, occurring in similar light conditions.
